# Outcomes of abdominal wall reconstruction in complex ventral hernia patients: a single institution based prospective study

**DOI:** 10.3389/fsurg.2025.1634748

**Published:** 2025-07-10

**Authors:** Bhawani Khanal, Abhijeet Kumar, Ashok Panta, Susmita Khadka Chhetri, Parbatraj Regmi, Vijay Pratap Sah, Bikash Kumar Sah, Davide Lomanto, Rakesh Kumar Gupta

**Affiliations:** ^1^ Department of General Surgery and MIS Unit, B.P. Koirala Institute of Health Sciences, Dharan, Nepal; ^2^ Department of Surgery, Yong, Loo, Lin School of Medicine, National University Hospital, Singapore, Singapore

**Keywords:** abdominal wall reconstruction, botulinum toxin, complex ventral hernia, outcomes, prospective study

## Abstract

**Introduction:**

Complex ventral hernias, especially in patients with prior surgeries, large defects, or comorbidities, are associated with high rates of recurrence and complications such as infection, pain, and loss of abdominal domain. This study aims to contribute to developing standardised management strategies.

**Methods:**

A prospective study was conducted at BP Koirala Institute of Health Sciences over two years, involving 38 patients undergoing abdominal wall reconstruction for complex ventral hernias. Preoperative assessment included NCCT and selective use of botulinum toxin for optimisation. Surgical approaches were individualised. Data on demographics, hernia characteristics, surgical technique, operative time, complications, hospital stay, recurrence, and chronic pain were collected and analysed.

**Results:**

Of 88 ventral hernia cases, 44 were complex; 38 underwent repair. Most were incisional hernias located at M2–M5, with a mean defect size of 7.1 ± 2.9 cm. Mean operative time was 154.8 ± 51.6 minutes. Complications included seroma (15.6%), SSI (15.6%), hematoma (5.3%), and enterotomy (5.3%). Average hospital stay was 2.8 ± 1.2 days; activity resumed in 7.5 ± 2.9 days. At 2-year follow-up, recurrence was seen in 5.2%.

**Conclusion:**

Tailored individualised planning is crucial in complex abdominal reconstruction due to patient and defect variability, making standard techniques impractical.

## Introduction

As long as surgeons utilise midline fascial incisions to access the abdominal cavity, incisional hernias will remain an unavoidable problem. There is an inherent risk of hernia formation that is quoted as high as 20%, and increases with wound infection and patient factors, such as morbid obesity, tobacco abuse, and immunosuppression (1).

The definition of ventral hernia is inconclusive. However European Society of Hernia has defined any hernia as a complex hernia that meets the following criteria (2).

1. size and location

Hernia with width >10 cm.

Hernia in unusual locations.

Loss of domain of >20%.

2. contamination and soft tissue condition (3)
3. patient's history and risk factors; Recurrent hernia with use of mesh previously, comorbidities, history of abdominal dehiscence
4. clinical scenarios: Emergency operation with bowel resection

Multiple hernia defects, Complex or recurrent abdominal wall defects, may be the result of a failed prior attempt at closure, trauma, infection, radiation necrosis, or tumour resection (3). The reconstruction of complex abdominal wall defects can often pose a significant challenge to surgeons and their patients. Left untreated, complex abdominal defects may result in significant physical discomfort, functional restrictions, loss of domain and in some cases, intestinal obstruction (4).

Decisions regarding technique for abdominal wall reconstruction were based on an assessment of the defect by location, extent (layers involved), and aetiology. Reconstructive options include direct tissue closure, prosthetic mesh, local advancement or regional flaps, distant flaps, or combined flap and mesh (3). The ultimate goals of abdominal wall reconstruction are to restore functional integrity, to provide support, to protect the abdominal viscera, and to minimise complications and recurrences (5).

Surgical site infection (SSI), Surgical site occurrence (SSO), Chronic pain, recurrence, mesh infection, and seroma formation rate are still the major concerns in all types of repairs. The complex ventral hernia is one of the major problems in our hospital, causing distress to patients and posing strong challenges to the surgeon.

This study aims to evaluate both early and long-term outcomes of abdominal wall reconstruction in patients with complex ventral hernias. The primary outcomes assessed include the incidence of hernia recurrence and chronic pain. The secondary outcomes included total hospital stay, hematoma, seroma, surgical site infection, and enterocutaneous fistula formation. The findings of this study are expected to help in formulating a specific protocol to improve management and outcomes in these challenging cases.

## Methodology

### Study design and setting

This prospective observational study was conducted at BP Koirala Institute of Health Sciences, a tertiary care centre, between May 1, 2022, and April 31, 2024.

### Study population

All patients with the diagnosis of complex ventral hernia fulfilling the criteria of the European Hernia Society and who gave consent for surgery were admitted to the surgery ward from the surgery outpatient department (OPD). A detailed history of the patient was taken and filled in the proforma. Hernia was classified according to the European Hernia Society classification of primary ventral hernia (6). The patient characteristics for the complex ventral hernia has been shown in Table 1.

**Table 1:** Patients characteristics with complex ventral hernia.

Character that defines complex ventral hernia	Frequency (proportion)
Width >10 cm	4
Loss of domain of >20%	29
Contamination and soft tissue condition	10
Recurrent hernia with history of mesh repair	2
Comorbidities (diabetes, hypertension, COPD)	12
Exploratory laparotomy with or without bowel resection	20

### Preoperative assessment and intervention

Preoperative need for NCCT of abdomen and pelvis was decided as per the clinical assessment of the team of surgeons involved in treating the patients. Use of Inj. Botulinum toxin was used to infiltrate the abdominal muscle to tackle the loss of domain, and it was based on the team of surgeons involved in treating the patients. Those groups of patients in whom the toxin was used were posted for surgery after 1 month of receiving the toxin.

Those patients who didn't need toxin injection to tackle loss of domain were posted for surgery after getting PAC fitness from the anaesthesia team. Patients who underwent surgery were counselled about the detailed procedure, likely outcomes, and unfavourable outcomes of the surgery, and those who gave consent were posted for surgery.

### Surgical procedure

Surgery was performed under general anaesthesia. Preoperative injection of ceftriaxone antibiotic was given at the time of induction. Painting and draping were done. The team of surgeons decided pre-operatively whether the patient needed simple primary closure, component separation techniques, peritoneal flap mesh hernioplasty, and proceeded accordingly (Table 2). The standard technique was followed in all surgeries.

**Table 2:** Techniques used in abdominal wall reconstruction.

Techniques	No of patients (%)
B/L TAR	12 (31.6%)
U/L TAR	9 (23.7%)
PFH	7 (18.4%)
PFH + U/L TAR	6 (15.8%)
LVHR	4 (10.5%)

In all procedures, lightweight Prolene mesh was placed except in cases of laparoscopic hernia repair where composite mesh was used. But the site of placement of the mesh and the size of the mesh were determined by the operating team. In all procedures Romovac suction drain was kept except in those cases who underwent laparoscopic ventral hernia repair (IPOM).

Skin was closed with a skin stapler, and a compressive dressing was applied. After completion of the procedure, patients were shifted to the surgery observation ward.

### Postoperative care

Patients were kept NPO till the patient had passed flatus. The patient was kept on maintenance fluid till the patient was orally allowed, in addition to inj. Ceftriaxone 1 gm twice a day, inj. Paracetamol 1 g four times a day, inj. Omeprazole 40 mg twice a day, and inj. Diclofenac sodium as required.

The surgical wound of the patient was observed on the second postoperative day. Patients were discharged once they could take care of themselves and were free of immediate complications. Drains were removed when the output was less than 30 ml daily for three consecutive days.

They were called for follow-up on the 10th postoperative day, and the clips were removed.

Those patients having complications were managed accordingly.

### Variables

- Patient demographics: Age, gender, body mass index (BMI, kg/m^2^).
- Hernia characteristics: The presence of pain, duration of hernia, and type of hernia as per EHS, reducibility and content of sac.
- Perioperative Variables: The assessed parameters included type of surgical technique, operative time (minutes), mesh type and intraoperative complications.
- Postoperative Variables: Postoperative pain was evaluated using the Visual Analogue Scale (VAS), ranging from 0 to 10, with pain scores recorded at 6, 12 and 24 hours postoperatively. Postoperative complications included wound morbidities, such as seroma, surgical site infection and port site haematoma. Duration of ileus, duration of hospital stay, duration for resuming routine activities and follow up for 2 years for recurrence and chronic pain.

## Results

During the study period, 88 patients presented with ventral hernia in our institute. Among them, 44 patients [38.63%] presented with complex ventral hernia, out of which only 38 patients underwent repair and were included in our study. Among 6 cases, 4 cases were not fit for general anaesthesia, and 2 were unwilling to undergo surgery and were excluded from the study.

The mean age of the patients in our study was 49.10 ± 11.92, ranging from 34 years to 78 years. The majority of the patients were male, 23 (60%). The mean BMI in our study was 23.87(23.87 ± 4.81 kg/m^2^).

Most of the patients had presented with a hernia for more than 6 years (6.73 ± 3.93). Almost all the hernias were incisional, and most were located at M2–5 (20%). The mean defect size in our study was 7.11 ± 2.89 cm. Pain at the hernia site was present in all the patients and most of them were reducible (60.5%).

Out of the 38 patients, 12 patients underwent B/L TAR (31.6%), 9(23.7%) patients underwent U/L TAR, 7(18.4%) patients underwent PFH, 6(15.8%) patients underwent PFH with U/L TAR and 4 (10.5%) underwent LVHR(IPOM). Omentum (60%), bowel (20%), omentum and bowel (20%) were the contents of the hernia.

The placement of polypropylene standard weight mesh was done in all the patients except one who underwent primary suture repair after resection-anastomosis of the gangrenous ileal segment.

The mean operating time was 154.77 ± 51.55 (90–230) minutes. The operation time was longer in those cases that needed additional procedure (216 ± 11.402 vs. 136.76 ± 44.05) minutes.

Bleeding from inferior epigastric vessels occurred in 2 (5.3%) patients, and omental bleeding in a single patient (2.6%), which was dealt with using electrocautery/harmonic sealing device.

Seroma was present in 6 patients (15.6%), which resolved after a few days. There were 2(5.3%) port site hematomas. There were 6(15.6%) cases of superficial surgical site infections, which were managed with daily dressing and antibiotics. Enterotomy occurred in 2(5.3%) patients, which was repaired primarily intraoperatively. All patients were kept NPO till the return of bowel sounds. The mean duration of ileus in our study was 35.40 ± 12.93 hours. Primary and secondary outcomes are represented in Tables 3, 4, respectively.

**Table 3:** Primary outcomes.

Primary outcomes	Number of patients (%)
Recurrence	
Early	1 (2.6%)
Late	1 (2.6%)
Chronic pain	0

**Table 4:** Secondary outcomes.

Secondary outcomes	Number of patients (%)
Hematoma	0
Seroma	6 (15.6%)
Wound infection	6 (15.6%)
Bleeding from inferior epigastric artery	2 (5.3%)
Enterocutaneous fistula	0
Enterotomy	2 (5.3%)

An additional analgesic was given to those patients who had a Visual Analogue Scale (VAS) score of more than 5. VAS was calculated at 6, 12, and 24 hours which was found to be 7.13 ± 0.98, 6.43 ± 0.56, 4.97 ± 0.92, respectively.

### Discharge and follow-up

The average length of hospital stay in our study was 2.83 ± 1.17 (range: 1–7) days. The average number of days after which they resumed their normal daily activities was 7.52 ± 2.9 (range: 4–13). All the patients were followed up for a minimum duration of 2 years. Few patients were followed up via telephonic conversation to assess the persistence of pain and recurrence of hernia. Only 4 patients (10.5%) had moderate pain requiring occasional intake of oral analgesics, though it was not severe enough to hamper their daily normal activities. There was 1 (2.6%) early recurrence (within a month) 1(2.6%) case of late recurrence noted till date.

## Discussion

The natural history of abdominal hernias has demonstrated that with time, patients’ quality of life will worsen with diminished social and physical functioning (7). More importantly, complex abdominal wall defects propagate additional morbidity and can result in substantial complications if left untreated. Thus, repair of such defects seems inevitable. Complex ventral hernia repairs can be frustrating reconstructive problems due to technical challenges and high postoperative complications. Chronic pain and recurrences after surgery have a detrimental effect on patient satisfaction. Thus, these defects require a distinct and more individualised, frequently interdisciplinary intervention beyond primary repair or the simple placement of mesh. Our experience in 38 patients reinforces the use of an individualised and tailored approach in complex ventral hernia repair for improving postoperative outcomes.

The patient demographics in our study are typical for the patient population that we see at our institution. As a tertiary academic referral centre, many of our patients are complicated. The majority of the cases were incisional hernias with larger defect sizes that were present for a prolonged period. This required extensive preoperative workup, planning and patient counselling. Luckily, our patients were not obese, with a mean of 23.87 kg/m^2,^ and we did not encounter preoperative enterocutaneous fistula in any of the cases.

Various laparoscopic and open techniques have been reported with the intention of decreasing recurrence rates in complex ventral hernia. Modified Rives-Stopa and posterior component separation techniques have demonstrated low recurrence rates below 10% (8, 9). However same technique might not be advisable for all patients. Satterwhite et al. described the use of various open techniques in 106 patients with complex ventral hernia, with a recurrence rate of 16% (5). The overall recurrence rate in our study was 6.66%, which seems acceptable.

Recurrence after hernia surgery has been shown to be associated with patient factors, including comorbidities (diabetes, Chronic Obstructive Pulmonary Disease, malnourishment), obesity, smoking as well as technical factors like method of defect closure, type and plane of mesh used, and postoperative infectious complications (10). Preoperative optimisation of these risk factors, including blood sugar control, smoking cessation, and other pulmonary and cardiac comorbidities, is required to decrease the chance of recurrence (11). Patients in our study were carefully optimised preoperatively, and weight reduction was advised in all patients. The low recurrence rate can be attributed to these factors, as all our patients had a desirable BMI of less than 30 kg/m^2^.

Use of synthetic mesh in all patients may be another factor for reducing the recurrence rate, as we used synthetic standard-weight polypropylene mesh in all open cases except for one patient who had enterotomy. We used the laparoscopic hernia repair technique whenever feasible, with the use of intraperitoneal composite mesh placement in 20% of the cases. This technique is reported to have a favourable outcome with a recurrence rate as low as 4.7% (12).

Pain after hernia surgery may be surgical pain, which usually subsides in 6 weeks to 6 months, or neuropathic pain, which may present as chronic pain sensation (13). We analysed immediate postoperative pain with visual analogue score in the 6-hour, 12-hour, and 24-hour postoperative period. Multimodal analgesia, including epidural, intravenous paracetamol, and NSAIDS, was given to mitigate acute pain. Increased pain was noted in the bilateral TAR group, which may be due to the large tissue dissection. Acute pain was well controlled with oral medication at the time of discharge. Laparoscopic repair had no significant advantage in terms of immediate postoperative pain than open repair.

Chronic pain after hernia repair has many potential causes and is likely multifactorial. Transfascial sutures in laparoscopic repair are assumed to cause acute as well as chronic postoperative pain, and removal of these sutures in an attempt to tackle chronic pain has also been reported with unpredictable outcomes (14). Prevalence of chronic pain after ventral hernia repair ranges from 7%–41% (15–17). Chronic pain was present in 2 patients who underwent TAR and 2 patients who underwent LVHR, who were all managed with oral NSAIDS taken on an as-needed basis. Preoperative pain has been reported to be a strong predictor of postoperative pain previously (18). On the contrary, in our study, almost all patients had some form of preoperative pain, which did not progress in the postoperative period. Surgical intervention or use of narcotics in the long term was not required in any case, reinforcing the wait-and-watch policy in management for chronic pain in ventral hernia surgery.

Surgical site occurrences are common in complex ventral hernia following open mesh repairs. Several factors affect surgical site occurrence, including diabetes, obesity, smoking, intraabdominal contamination, number of prior repairs, and size of defect. Previous studies have reported 13%–20% of surgical site infections and around 20% of seroma formation (5, 19, 20), which is in accordance with our study. One of the feared complications is mesh infection and the need for mesh excision, which was not present in our study. As expected, wound complications are higher in a contaminated wound than clean wound. Biological mesh is traditionally used for repair in contaminated cases, however, mesh was not used in one case of resection anastomosis in our study owing to the higher cost and availability.

Hospital stay, along with wound-related complications, is shown to be lower in laparoscopic cases as compared to open repair cases (21). Due to the complexity of procedures, repair of complex ventral hernia is thought to have higher days of hospital admission. Satterwhite et al. reported hospital stay from 1 to 78 days. Patients with longer than 14 days of hospital stay were associated with significant postoperative complications or required reoperation (5). In our study duration of hospital stay was 1–7 days. Absence of major postoperative complications like enterocutaneous fistulas or requirement of reoperations has played a major role in this favourable outcome.

### Strength

This study offers valuable insight into the management and outcomes of complex ventral hernia repairs in a real-world tertiary care setting in a developing country. A major strength lies in its prospective design, minimising the recall bias. The minimum two-year follow-up period strengthens the reliability of long-term outcome assessment, including recurrence and chronic pain. Furthermore, the use of standardised surgical techniques by a consistent surgical team and the inclusion of both open and laparoscopic repairs enhance the wide applicability.

### Limitation

Being a single-centre study and a small sample size of 38 may limit the generalisability of findings. In addition, follow-up via telephone in some patients might have led to underreporting of symptoms such as mild pain or minor complications. A potential limitation of our study is that all patients had a BMI of less than 30 kg/m², which may have contributed to the low recurrence rate observed and limits the generalisability of our findings to patients with higher BMI.

## Conclusion

Our study illustrates the need for preoperative optimisation and proper planning for complex abdominal reconstructions. Patients need to be managed with an individualised and tailored approach, and a “one size fits all” mentality cannot be applied when it comes to repairing these abdominal wall defects. Each patient presents with unique differences in comorbidities, tissue quality, defect sizes, and intraoperative bowel procedures that make standardisation of technique almost impossible. Future research should focus on larger multicentric trials and long-term outcomes to optimisethe management of complex ventral hernias.

## Data Availability

The original contributions presented in the study are included in the article/Supplementary Material, further inquiries can be directed to the corresponding author.
